# Single-cell and spatial transcriptome sequencing analysis reveals characteristics of a unique subpopulation in high-grade IDH-mutant astrocytoma

**DOI:** 10.1007/s13402-025-01139-5

**Published:** 2025-12-29

**Authors:** Wei Chen, Min Zheng, Jia-Yin An, Guo-Hao Huang, Jing-Peng Liu, Lin Yang, Peng Ren, Ting-Ting Wang, Jean-Philippe Hugnot, Sheng-Qing Lv

**Affiliations:** 1 Department of Neurosurgery, Xinqiao Hospital, Third Military Medical University (Army Medical University), Chongqing, China; 2 IGF, Univ Montpellier, CNRS, Inserm, Montpellier, France; 3 Jinfeng Laboratory, No. 313, Jinyue Road, Chongqing High-tech Zone, Chongqing, 401329 China; 4 Emergency Department, Naval Hospital of Eastern Theater, Zhejiang, China

**Keywords:** IDH-mutant astrocytoma, Single-cell sequencing, Spatial transcriptome sequencing, Malignant progression

## Abstract

**Introduction:**

(Isocitrate dehydrogenase) IDH-mutant astrocytoma is classified as World Health Organization (WHO) grade 2–4 and is second only to IDH wild-type glioblastoma in the incidence of adult glioma. However, few studies use single-cell and spatial transcriptome sequencing to analyze its malignant progression.

**Methods:**

Intraoperative navigation and yellow fluorescence visualization were utilized to accurately isolate high-grade (WHO grade 3–4) and low-grade (WHO grade 2) samples of IDH-mutant astrocytoma for single-cell and spatial transcriptome sequencing. By combining single-cell, spatial transcriptome, The Cancer Genome Atlas (TCGA), and The Chinese Glioma Genome Atlas (CGGA) data, analyses of survival, enriched pathways, transcription factors, intercellular communication, differentiation trajectories, and immune response were performed to identify the characteristics of a unique subpopulation of high-grade IDH-mutant astrocytoma.

**Results:**

Our single-cell RNA sequencing analysis identified a distinct subpopulation (Cluster 7) present in high-grade IDH-mutant astrocytoma, which was localized to the terminus of the pseudotime trajectory. Importantly, this cluster not only exhibited an immunosuppressive phenotype correlated with poor clinical prognosis, but also demonstrated significant enrichment in Developmental Biology and Calcium Signaling pathways. Furthermore, this subpopulation engaged in prominent ligand-receptor interactions, particularly through PTN_PTPRZ1 and MIF_CD74 pairs. Notably, comparative analysis revealed that high-grade astrocytoma displayed both quantitatively and qualitatively enhanced communication networks when compared to their low-grade counterparts.

**Conclusions:**

Our single-cell RNA sequencing analysis identifies a distinct tumor cell subpopulation present in high-grade (WHO grade 3–4) adult IDH-mutant astrocytoma. This cluster, which likely arises from malignant progression in adult astrocytoma, may provide new insights for developing therapeutic strategies against this clinically challenging disease.

**Supplementary Information:**

The online version contains supplementary material available at 10.1007/s13402-025-01139-5.

## Introduction

According to the 2021 WHO Classification of Tumors of the Central Nervous System, IDH-mutant astrocytoma was molecularly characterized by IDH mutations without 1p19q co-deletion and was classified as WHO grades 2–4 [1]. Among adult gliomas, IDH-mutant astrocytoma is second only to IDH wild-type glioblastoma in incidence, with an average survival time of 7–8 years, better than IDH wild-type glioblastoma and worse than oligodendroglioma [2–8].

The emergence of technologies such as single-cell and spatial transcriptome sequencing in recent years has allowed us to analyze the molecular characteristics and functions of different clusters of tumors. However, most studies on adult gliomas target IDH wild-type glioblastomas [9–11].

Through single-cell RNA sequencing, we identified a distinct *GSX1*^+^ tumor subpopulation specifically enriched in high-grade IDH-mutant astrocytoma (WHO grades 3–4), localized at the pseudotime trajectory terminus and associated with poor prognosis and immunosuppressive phenotype. This subpopulation exhibited marked pathway activation in REACTOME developmental biology and KEGG calcium signaling, with spatial transcriptomics validating its characteristic cell-cell communication patterns, particularly through dominant PTN_PTPRZ1 and MIF_CD74 interactions. Comparative analyses revealed significantly enhanced communication network complexity in high-grade versus low-grade tumors. Collectively, these results support a role for *GSX1*^+^ cluster in IDH-mutant astrocytoma malignant progression, with potential implications for developing targeted therapies against this aggressive tumor subtype.

## Materials and methods

### Sample cohort and selection criteria

We performed 10x single-cell RNA sequencing on matched high-grade (WHO grade 3–4, *n* = 3) and low-grade (WHO grade 2, *n* = 3) IDH-mutant astrocytomas, with additional spatial transcriptomics from one tumor junction area of high-grade and low-grade IDH-mutant astrocytoma. High-grade cases met: (1) age > 18 years (2), MRI contrast enhancement (3), intraoperative yellow fluorescence, and (4) pathological confirmation of IDH mutation with 1p/19q intact status. Low-grade tumors exhibited: (1) age > 18 years (2), non-enhancing MRI (3), absent fluorescence, and (4) equivalent molecular confirmation. All cases were histopathologically validated per the 2021 WHO CNS guidelines (1).

### Single-cell sequencing and upstream data processing

Fresh surgical samples were dissected into 2–4 mm fragments on ice, followed by tissue dissociation, cell counting, quality control, and library preparation. Sequencing reads were demultiplexed and aligned to the reference genome using the 10x Genomics Cell Ranger pipeline (v7.1) with default parameters, which performs alignment, barcode/UMI counting, and generates feature-barcode matrices. Initial quality control included doublet removal (Scrublet, expected_doublet_rate = 0.07) and filtering via Seurat to retain high-quality cells (genes > 200, UMIs > 400, mitochondrial reads < 20%) [12].

### Spatial transcriptome sequencing and upstream data processing

Tumor samples preserved in OCT were processed through RNA quality assessment, fixation, H&E staining, destaining, probe hybridization, RNA digestion, probe release, reverse transcription, and library preparation. Libraries were purified (SPRIselect), quality-checked (Bioanalyzer), and sequenced on an Illumina NovaSeq platform (PE150). Primary quality control of FASTQ files included: (1) discarding reads with > 3 N bases (excluding paired reads) (2), removing read pairs if ≥ 20% of bases had Q < 5, and (3) trimming adapter sequences (minimum 8 bp match). Reads were aligned to the probe set using Space Ranger Count, with confident mappings (paired-probe matches) retained after filtering homologous genes [11].

### Double-cell removal, quality control, and single-cell sample integration

In the downstream data analysis, the R package DoubletFinder (v2.0.4) was applied for double cell removal according to the instructions (https://github.com/ddiez/DoubletFinder). In short, double cell removal was exercised for each sample, DoubletRate = ncol(number of cells)*8*1e-6. Calculated double-cell ratios were corrected using homologous double-cell ratios, pcs = 1:20, pN = 0.25 [13]. The data was then integrated, and QC was performed on the following criteria: nFeature_RNA > 300 & nFeature_RNA < 6000 & percent.mt < 10 & percent.HB < 1 & nCount_RNA < 100,000. De-batch effects were then performed using the harmony package [14].

### Seurat pipeline

Single-cell RNA-seq data were processed using Seurat (v5.1.0) following standard workflows. Data normalization (LogNormalize, scale factor = 10,000) was followed by identification of highly variable genes (vst method, nfeatures = 3,000) and scaling. Dimensionality reduction included PCA (*n* = 20 PCs confirmed by elbow plot) and UMAP (dims = 1:20, harmony-corrected). Clustering (resolution = 0.4, dims = 1:20) and differential expression analysis (FindAllMarkers, est.use = ‘wilcox’, only.pos = T, min.pct = 0.4, logfc.threshold = 1) were performed to characterize cell populations [12].

### TCGA, CGGA data processing

Glioma RNA-seq counts data and clinical information were obtained from TCGA (via UCSC Xena, *n* = 223, IDH-mutant astrocytomas: 114 grade 2, 100 grade 3, 9 grade 4) and CGGA (*n* = 206, IDH-mutant astrocytomas from 693 glioma dataset: 70 grade 2, 101 grade 3, 35 grade 4), with samples stratified by IDH mutation and 1p/19q intact status [15, 16]. Data integration and batch correction were performed using IOBR (v0.99.0; remove_batcheffect function), followed by counts-to-TPM conversion (count2tpm function) [17].

### Functional enrichment analysis

Functional enrichment analysis was performed at three biological scales: (i) differential gene set analysis using Gene Set Enrichment Analysis (GSEA) with MSigDB collections [18, 19], (ii) single-cell pathway activity quantification via AUCell (https://github.com/aertslab/AUCell), and (iii) spatial transcriptomic functional patterns assessed through Seurat’s AddModuleScore with default parameters.

### SCENIC

Transcription factor regulatory networks were reconstructed using SCENIC (v1.3.2) with hg38 reference databases (refseq-r80__10kb_up_and_down_tss.mc9nr.feather and refseq-r80__500bp_up_and_100bp_down_tss.mc9nr.feather). Gene expression matrices were filtered (minCountsPerGene = 3 × 0.01 × ncol(exprMat); minSamples = ncol(exprMat) × 0.01). The most and least active transcription factors were filtered based on regulon activity scores [20].

### InferCNV, Monocle2, velocity

Copy number variation analysis was performed using inferCNV (https://github.com/broadinstitute/inferCNV/wiki) with human.gene.positions as the genomic reference, implementing the run function (cutoff = 0.1, denoise = TRUE, hclust_method=’ward.D2’) and T cells as the reference population [21]. Pseudotime trajectory reconstruction was conducted via monocle (v2.32.0) by: (i) creating CellDataSet objects (newCellDataSet), (ii) calculating normalization factors (estimateSizeFactors), and (iii) estimating gene dispersions (estimateDispersions) [22]. RNA velocity was estimated from integrated loom files using scVelo (v0.3.2) [23].

### Cellchat

Intercellular communication analysis was performed using CellChat (v1.6.1) following the developer’s protocol. The pipeline comprised: (i) object initialization with createCellChat, (ii) identification of significantly overexpressed ligand-receptor pairs via identifyOverExpressedInteractions (*P* < 0.05), (iii) probabilistic interaction modeling using computeCommunProb, and (iv) filtration of low-confidence interactions (filterCommunication; min.cells = 5). Resultant networks were visualized through multiple modalities, including global connectivity patterns (netVisual_circle, netVisual_aggregate), interaction-specific diagrams (netVisual_heatmap, netVisual_individual), ligand-receptor expression mapping (netVisual_bubble), and molecular context visualization (plotGeneExpression) [24].

### Immune response analysis

To achieve robust statistical power, we integrated 429 IDH-mutant astrocytoma samples from the TCGA and CGGA databases. Tumor Immune Dysfunction and Exclusion (TIDE) scores were computed for each case using the official web portal (v2.0; http://tide.dfci.harvard.edu/) [25]. Gene Set Variation Analysis (GSVA) was then performed using cluster 7 signature genes as input. Samples were dichotomized into high- and low-score groups based on the median GSVA enrichment score [26], followed by comparative analysis of immunotherapy response potential.

### Process of spatial transcriptome analysis

Spatial transcriptomic data processing, including quality control, dimensionality reduction, and clustering, was performed using Seurat (v5.1.0) following the developer’s standardized pipeline. Gene set activity scores were quantified via the AddModuleScore algorithm, with spatial expression patterns visualized using SpatialFeaturePlot. Gene set variation analysis (GSVA) was employed to quantify gene set enrichment scores. For spatial deconvolution, the Robust Cell Type Decomposition (RCTD) algorithm was implemented to integrate single-cell RNA sequencing (scRNA-seq) with spatial transcriptomic data [27]. Spatial co-localization patterns were analyzed using mistyR (v1.14.0), a computational framework for multi-view tissue characterization [28]. Cell-cell communication networks were inferred through stLearn (v1.1.1), incorporating spatial proximity constraints into ligand-receptor interaction modeling [29].

### Cell lines and cell culture

The human Astrocytoma cell lines (LGG336, LGG85) were obtained from Montpellier University Hospital (CHUGui de Chauliac), following the procedures previously described [30]. LGG336 and LGG85 cells were cultured in Dulbecco’s Modified Eagle’s medium F12 (#21331-020, Gibco) supplemented with L-glutamine (#25030081, Gibco), N2 (#17502048, Gibco), B27 (#12587010, Gibco), ciprofloxacin (#PHR1044, Sigma), gentamicin (# HY-A0276A, MCE), fungin (#ant-fn-1, Invivogen), heparin (#H3149, Sigma), EGF (#AF-100-15, Peprotech), and FGF2 (#AF-100-18B, Peprotech), with cultures incubated at 37 °C in a humidified 5% CO₂ atmosphere.

### Western blot

The antibodies were used for Western blotting: Gsh1 (Gsh1 Rabbit pAb, bs-11612R,1:500), PDE1C (PDE1C Polyclonal antibody,13785-1-AP, 1:500), beta-Actin (beta-Actin (8H10D10) Mouse mAb). Cells were lysed in RIPA Lysis Buffer (P0013B, Beyotime). Protein samples (10 mg per lane) were separated by SDS-PAGE electrophoresis and immunoblotted on polyvinylidene fluoride (PVDF) membranes and incubated with the primary antibody at 4˚C overnight. After washing, membranes were incubated with the secondary antibody (beta-Actin to anti-Mouse (ABflo^®^ 488-conjugated Goat anti-Mouse IgG (H + L) (AS037)). After incubation with rabbit primary antibodies against Gsh1 and PDE1C, samples were treated with HRP-conjugated anti-rabbit IgG secondary antibody at room temperature for 60 min, followed by ECL detection using a CCD camera. Quantification was performed using ImageJ software.

### Immunohistochemistry (IHC)

Immunohistochemistry was performed on 4-µm paraffin-embedded astrocytoma sections. After dewaxing and rehydration, antigen retrieval was conducted in citrate buffer (pH 6.0) at 95 °C for 30 min. Endogenous peroxidase was blocked with 3% H₂O₂ (25 min, RT), followed by 3% BSA blocking (30 min). Sections were incubated with primary antibodies (anti-PDE1C 1:500, anti-SOX10 1:50) overnight at 4 °C, then with HRP-conjugated secondary antibody (50 min, RT). DAB development was monitored microscopically, followed by hematoxylin counterstaining. Sections were dehydrated and mounted for imaging.

### Co-Immunofluorescence staining

Immunofluorescence was performed on 4-µm sections cut with a Leica RM2016 microtome. Sections were dewaxed using a Donatello dehydrator (DIPATH) and rehydrated through an ethanol series. Antigen retrieval was conducted in citrate buffer (pH 6.0) using a Galanz microwave oven (P70D20TL-P4) at 95 °C for 30 min. After peroxidase blocking (3% H₂O₂, 25 min), sections were permeabilized with 0.5% Triton X-100 and blocked with 10% serum. Primary antibodies (anti-Gsh1, bs-11612R, 1:500; anti-IDH1 R132H, H09, Dianova 1:100) were incubated overnight at 4 °C in a Haier BCD-192TGN icebox. TSA amplification was performed after HRP-secondary antibody incubation (50 min) on a Servicebio DS-2S100 decolorizing shaker. Fluorescent secondaries (Alexa Fluor^®^ 488/594, 1:500) were applied for 50 min, followed by DAPI counterstaining (10 min). Slides (Servicebio G6012) were mounted with coverslips (Jiangsu Shitai 10212432 C) using antifade medium and imaged on a Nikon Eclipse C1 microscope with consistent exposure settings.

### R, Python, and statistical methods

All analyses were performed using R (v4.4.0) and Python (v3.10). Data are presented as mean ± SEM unless noted otherwise in figure legends. Normality was assessed by the Shapiro–Wilk test. Parametric data were analyzed using Student’s t-test or ANOVA (one-way/two-way), with Tukey’s HSD test for multiple comparisons. Non-parametric data were evaluated with the Mann-Whitney test, except for large samples (*n* > 30/group) where Welch’s t-test was applied. Survival analyses used log-rank (Mantel–Cox) tests, and categorical data were assessed by Fisher’s exact test. Significance thresholds were set at *P* < 0.05.

## Results

### Identification of a *GSX1*^+^ tumor subpopulation associated with poor prognosis in high-grade IDH-mutant Astrocytoma

**Fig. 1:**
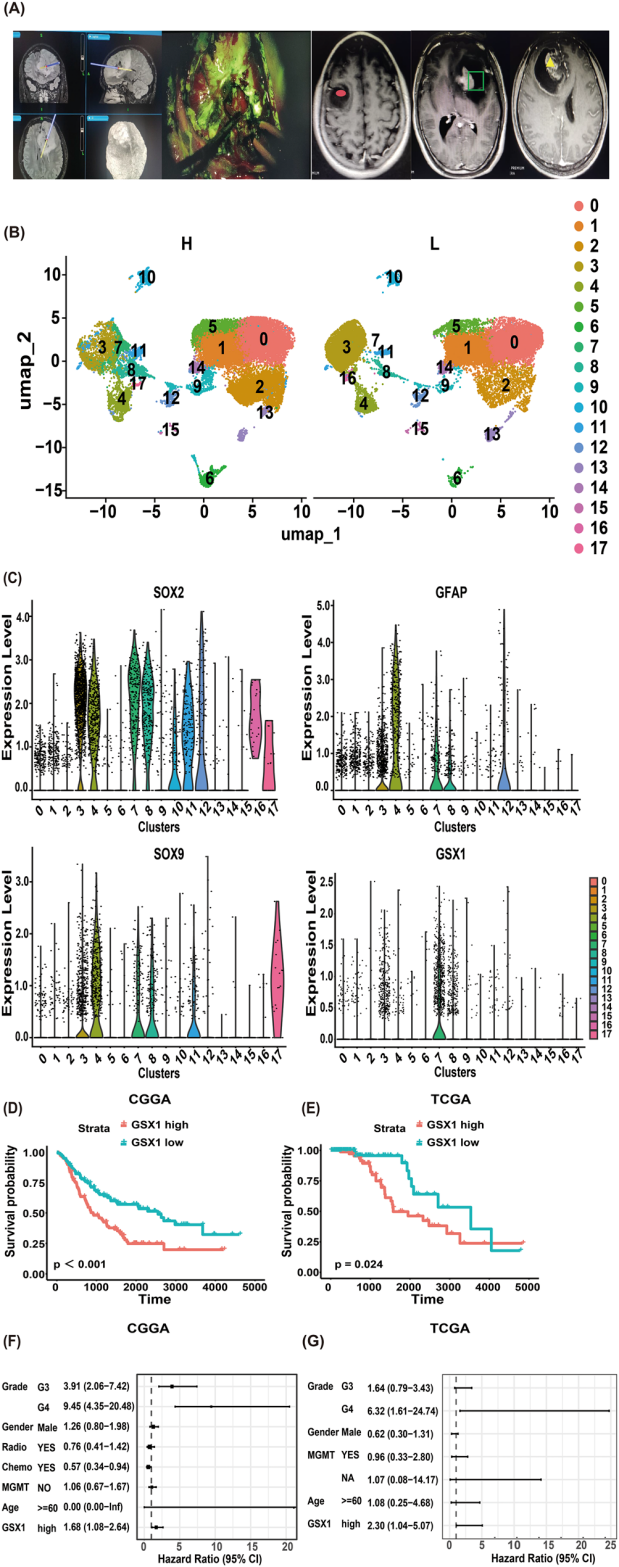
Identification of a *GSX1*^+^ tumor subpopulation associated with poor prognosis in high-grade IDH-mutant astrocytoma. (**A**) Schematic representation of tumor sampling regions: red spots denote low-grade areas, yellow triangles indicate high-grade areas, and green boxes demarcate mixed high- and low-grade zones. (**B**) DimPlot of high-grade and low-grade tumor samples. (**C**) VlnPlot of *SOX2*,* GFAP*,* SOX9*, and *GSX1*. (**D**) *GSX1*-high vs. *GSX1*-low survival in CGGA (*n* = 206). (**E**) TCGA cohort (*n* = 223). Groups stratified by median expression. Log-rank (Mantel–Cox) test. (**F**) Multivariate analysis identified high *GSX1* expression as an independent prognostic factor in both CGGA (HR = 1.68) and (**G**) TCGA (HR = 2.30) cohorts. H: high-grade IDH-mutant astrocytoma. L: low-grade IDH-mutant astrocytoma. Radio: Radiotherapy. Chemo: Chemotherapy. MGMT: MGMT promoter methylation. NA: Not available

Utilizing intraoperative neuronavigation and yellow fluorescence guidance, we achieved precise tumor resection, with distinct pathological regions demarcated as follows: red spots (low-grade areas), yellow triangles (high-grade areas), and green boxes (mixed high/low-grade zones) (Fig. 1A). Cell clusters were annotated based on canonical markers from established literature [31]: tumor cells (SOX2^+^SOX9^+^GFAP^+^), M1-like MDM (CD68^+^P2Y12^−^CD163^−^), M2-like MDM (CD68^+^P2Y12^−^CD163^+^), M1-like MG (CD68^+^P2Y12^+^CD163^−^), M2-like MG (CD68^+^P2Y12^+^CD163^+^), T cells (CD68^−^CD3^+^), monocytes (CD14^+^ and/or CD16^+^), endothelial cells (CD31^+^). Unsupervised clustering identified tumor cell-enriched clusters [3, 4, 7, 8, 11, 12, 16, 17], microglia (0, 1, 5, 14), macrophages [2], monocytes [9], T cells [6], endothelial cells [15], and undefined populations [10, 13]. Notably, a distinct tumor subpopulation (cluster 7, *n* = 1,146 cells) was exclusively detected in high-grade samples (Fig. 1B). Differential gene expression analysis (*FindAllMarkers*) was subsequently performed to identify cluster-specific markers. The DotPlot visualization (Supplementary Fig. 1) delineated canonical marker genes for each cell type, as referenced in prior studies [31]. Cluster 7 was identified as a distinct subpopulation characterized by co-expression of *GSX1*,* SOX2*,* SOX9*, and *GFAP* (Fig. 1C). Furthermore, independent validation in IDH-mutant astrocytoma datasets confirmed *GSX1* expression within the tumor cell population (Supplementary Fig. 2; Single Cell Portal: https://singlecell.broadinstitute.org/) [32]. Elevated *GSX1* expression correlated with poorer prognosis in both the CGGA (Fig. 1D) and TCGA cohorts (Fig. 1E). Multivariate analysis confirmed *GSX1* as an independent prognostic factor in these datasets (CGGA: Fig. 1F; TCGA: Fig. 1G). Although Cluster 17 was uniquely observed in high-grade astrocytoma, its limited cell count (*n* = 68) precluded further investigation in this study.

### Functional enrichment of cluster 7

**Fig. 2:**
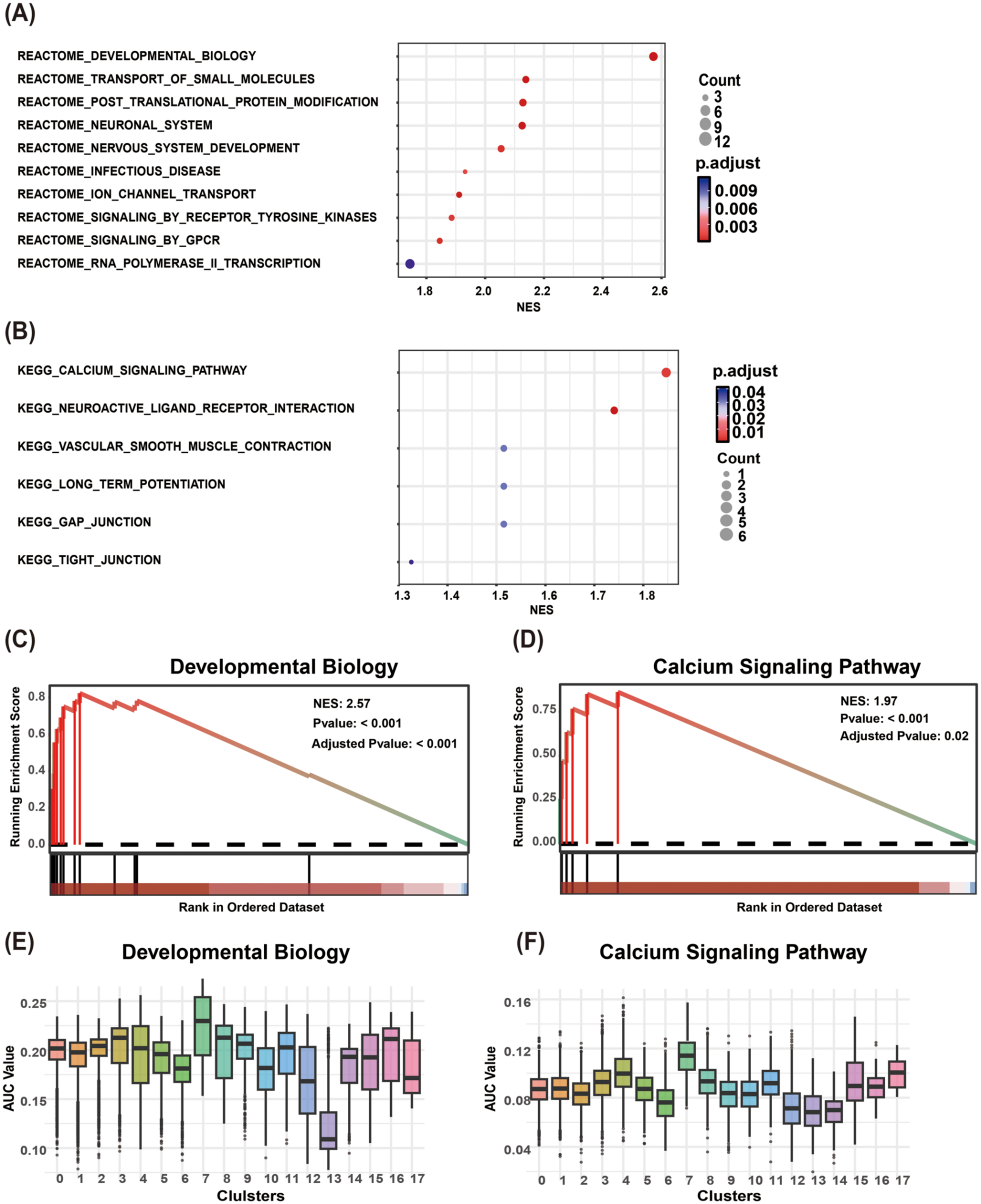
Functional enrichment of cluster 7. (**A**) Top 10 enriched pathways from the REACTOME database by GSEA. (**B**) Top 6 enriched pathways from the KEGG database by GSEA. (**C**) GSEA enrichment plot for REACTOME_DEVELOPMENTAL_BIOLOGY. (**D**) GSEA enrichment plot for KEGG_CALCIUM_SIGNALING_PATHWAY. (**E**) Single-cell pathway activity comparison of REACTOME_DEVELOPMENTAL_BIOLOGY by AUCell scoring. (**F**) Single-cell pathway activity comparison of KEGG_CALCIUM_SIGNALING_PATHWAY by AUCell scoring. KEGG: Kyoto Encyclopedia of Genes and Genomes

Using the FindAllMarkers function (parameters: min.pct = 0.25, test.use = “wilcox”, logfc.threshold = 0.25), we identified differentially expressed genes (DEGs) between cluster 7 and all other clusters. Subsequent Gene Set Enrichment Analysis (GSEA) revealed that cluster 7 was most significantly enriched in the REACTOME_DEVELOPMENTAL_BIOLOGY pathway (Fig. 2A, C) and the KEGG_CALCIUM_SIGNALING_PATHWAY (Fig. 2B, D). Key genes driving the REACTOME_DEVELOPMENTAL_BIOLOGY enrichment included *SOX10*,* HOXD3*,* TFAP2C*,* LHX2*,* COL9A1*,* IRX1*,* HOPX*,* SIX1*,* BOC*,* CAPN2*, and *COL9A3*, while the KEGG_CALCIUM_SIGNALING_PATHWAY was enriched for *PDE1C*,* ATP2B2*,* ADCY1*,* GRM5*,* TACR1*, and *ADRA1A*. To validate the activity of these pathways at single-cell resolution, we employed AUCell scoring, which demonstrated significantly higher pathway activity in cluster 7 compared to other clusters (Fig. 2E- F).

### Experimental validation of GSX1-Enriched subpopulation in Astrocytoma

**Fig. 3:**
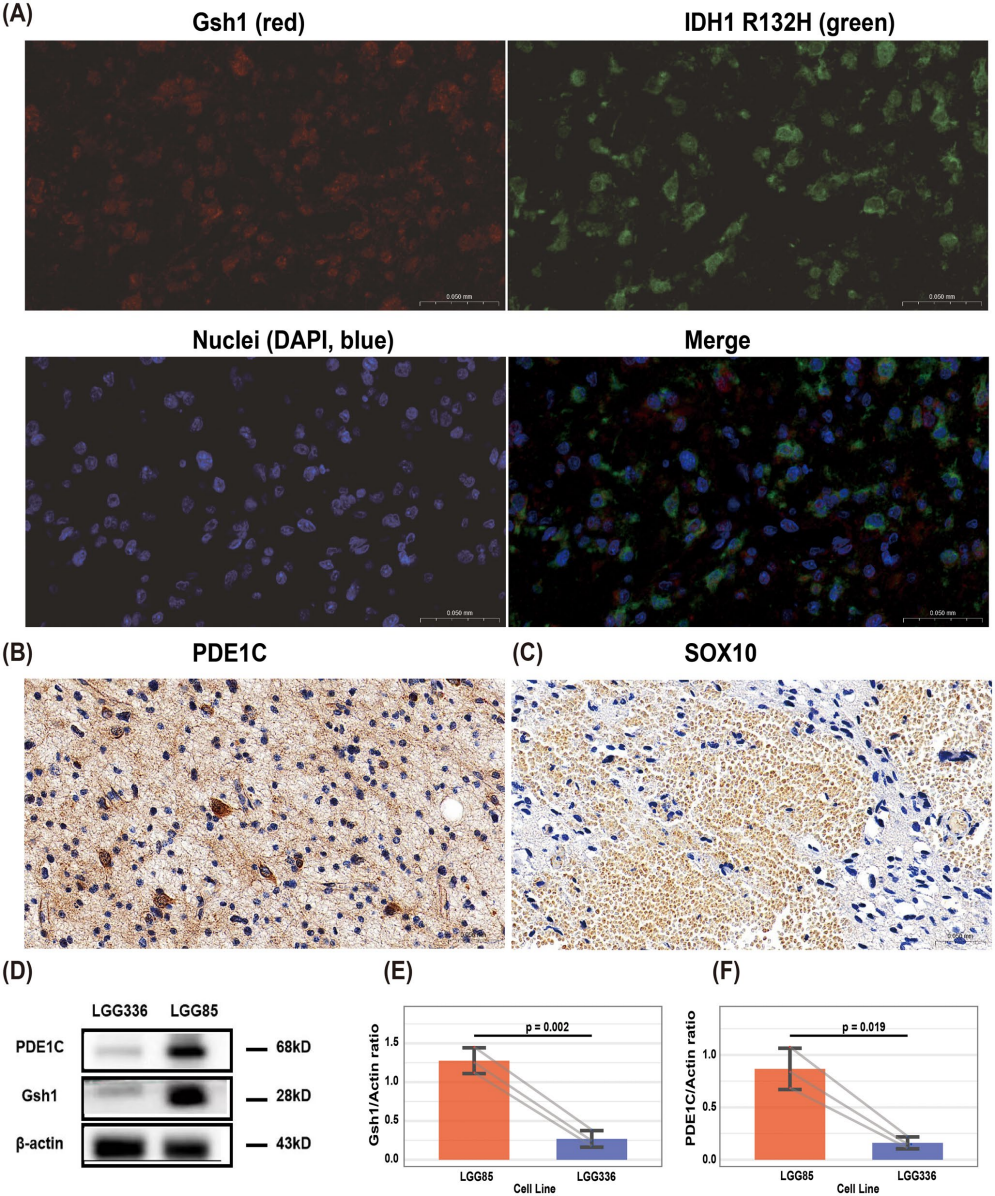
Experimental validation of GSX1 expression in astrocytoma. (**A**) Fluorescence co-localization of Gsh1 (red) and IDH1 R132H mutant (green) in tumor cells. Nuclei counterstained with DAPI (blue). 63x. (**B**) Representative immunohistochemical staining of PDE1C in high-grade IDH-mutant astrocytoma specimens. 40x. (**C**) Representative immunohistochemical staining of SOX10 in high-grade IDH-mutant astrocytoma specimens. 40x. (**D-F**) Western blot analysis of Gsh1 and PDE1C protein levels in high-grade (LGG85, WHO grade 4) versus low-grade (LGG336, WHO grade 2) IDH-mutant astrocytoma cell lines

To validate our findings, we performed experiments using IDH-mutant astrocytoma cell lines LGG85 (WHO grade 4, high-grade) and LGG336 (WHO grade 2, low-grade). Fluorescence co-localization assays revealed spatial co-expression of Gsh1 with the *IDH1* R132H mutant (Fig. 3A). Moreover, key downstream effectors of these pathways—PDE1C (Calcium Signaling Pathway) and SOX10 (Developmental Biology)—exhibited distinct expression patterns, as demonstrated by representative immunohistochemical staining (Fig. 3B–C). Western blot analysis confirmed significantly higher Gsh1 and PDE1C protein expression in high-grade astrocytoma (LGG85) compared to low-grade astrocytoma (LGG336) (Fig. 3D-F). These results collectively demonstrate: [1] the existence of a GSX1 (Gsh1)-enriched subpopulation co-localizing with tumor cells; [2] a grade-dependent expression pattern of Gsh1 and PDE1C.

### SCENIC analysis of transcription factor regulation in cluster 7

**Fig. 4:**
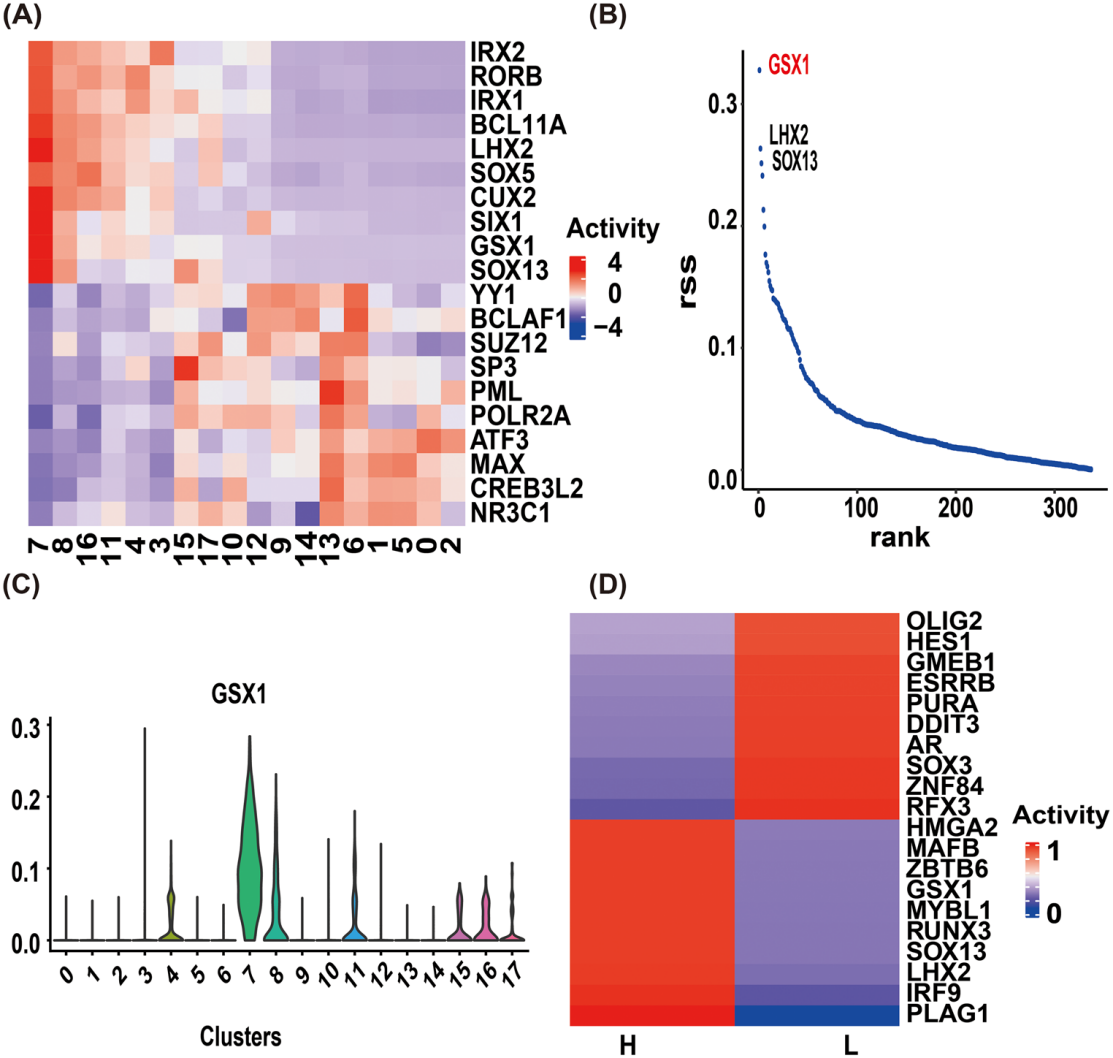
SCENIC analysis of transcription factor regulation in cluster 7. (**A**) Heatmap showing the activity of the top 10 most and least active transcription factors (TFs) in cluster 7 across all clusters. (**B**) Visualization of all TFs identified in cluster 7. (**C**) Violin plot depicting GSX1 TF activity distribution across clusters. (**D**) Top 20 differentially active TFs between high-grade and low-grade IDH-mutant astrocytoma. H: high-grade IDH-mutant astrocytoma; L: low-grade IDH-mutant astrocytoma

We performed SCENIC analysis to investigate transcription factor (TF) activity in cluster 7. The top 10 most/least active TFs were identified, and their activity patterns across all clusters revealed two distinct groups: tumor cell clusters [3, 4, 7, 8, 11, 16] and non-tumor clusters (13, 6, 1, 5, 0, 2) (Fig. 4A). Among all TFs in cluster 7, GSX1 (red) exhibited the highest activity (Fig. 4B), consistent with its reported role in progenitor cell proliferation, differentiation, and neural development. Cluster-specific analysis confirmed GSX1’s predominant activity in cluster 7 (Fig. 4C). Comparative analysis of the top 20 differentially active TFs between IDH-mutant high-grade and low-grade astrocytoma revealed distinct regulatory profiles (Fig. 4D).

### Copy number variation and tumor subcluster dynamics

**Fig. 5:**
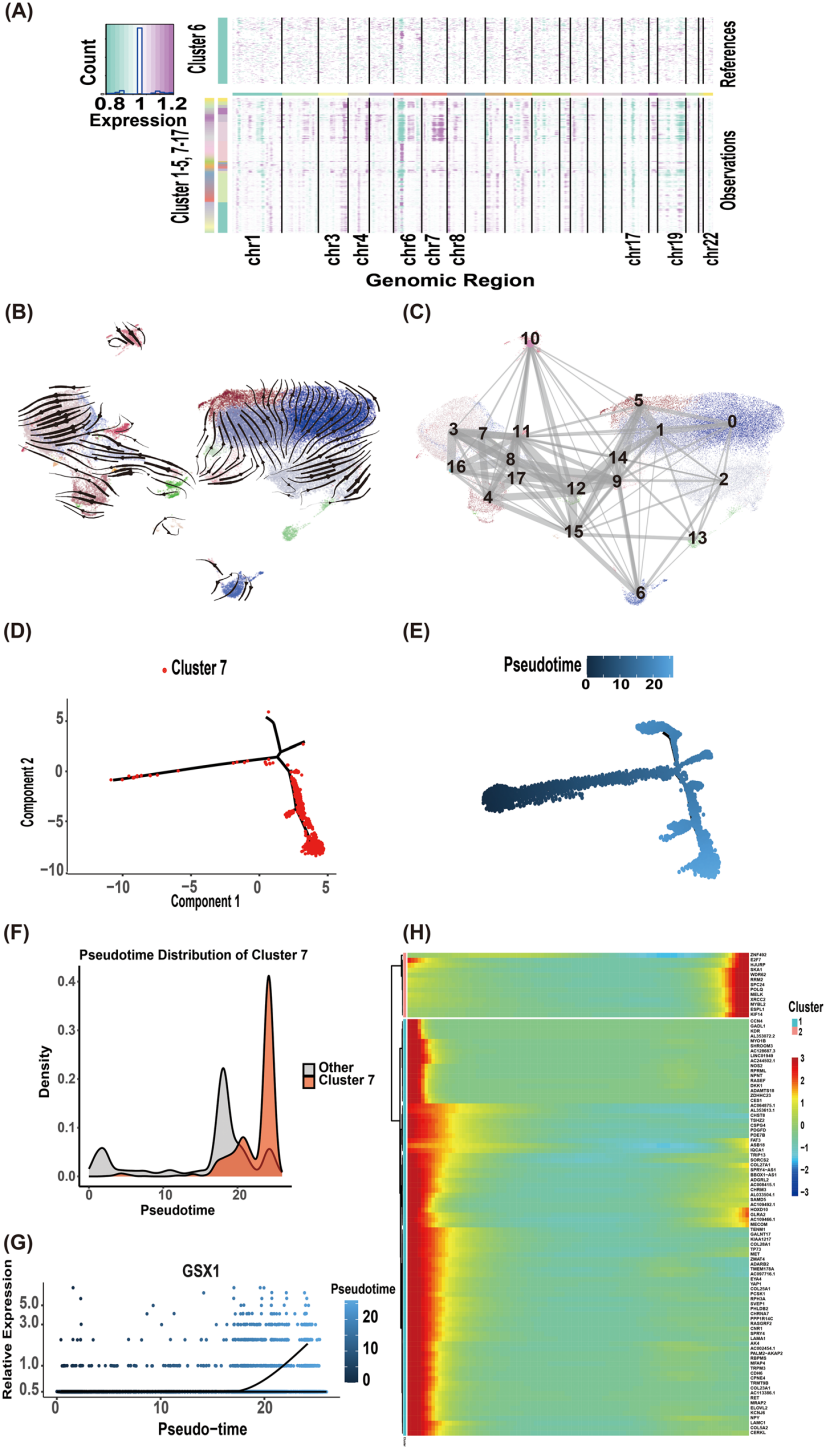
Copy Number Variation and tumor subcluster dynamics. (**A**) InferCNV analysis revealed genome-wide copy number variations across all single cells. (**B**–**C**) RNA velocity analysis delineated dynamic transitions between subpopulations. (**D**–**F**) Pseudotime trajectory reconstruction of all tumor subgroups positioned cluster 7 at the terminal state. (**G**) *GSX1* expression exhibited a significant positive correlation with pseudotime progression in cluster 7. (**H**) Pseudotime-dependent expression trends of cluster 7 signature genes segregated into functional modules: proliferation-associated (*MYBL2*,* E2F7*) and invasion-associated (*CCN4*,* MET*)

InferCNV analysis using T cells as reference revealed significant copy number amplifications (chromosomes 7, 8, 3, 4) and deletions (chromosomes 17, 19) across clusters (Fig. 5A).

To delineate tumor subcluster relationships, RNA velocity and Monocle2 pseudotime analysis were integrated. Velocity analysis demonstrated directional progression of clusters 4, 8, 11, and 17 toward cluster 7 (Fig. 5B-C), while Monocle2 positioned cluster 7 at the terminal pseudotime state (Fig. 5D-F). *GSX1* expression in cluster 7 increased significantly along pseudotime (Fig. 5G). Then we performed the cell trajectory analysis of the signature genes of cluster 7 and found that genes such as *MYBL2*,* E2F7* were clustered into one group. On the other hand, *CCN4* and *MET* were clustered into one group (Fig. 5H). Many studies on *MYBL2*,* E2F7*,* CCN4*, and *MET* genes promote tumor progression by promoting tumor proliferation, differentiation, and migration [10, 33–35], which may also contribute to the malignant progression of IDH-mutant astrocytoma.

### Cluster 7 communication networks and grade-specific interactions in IDH-mutant Astrocytoma

**Fig. 6:**
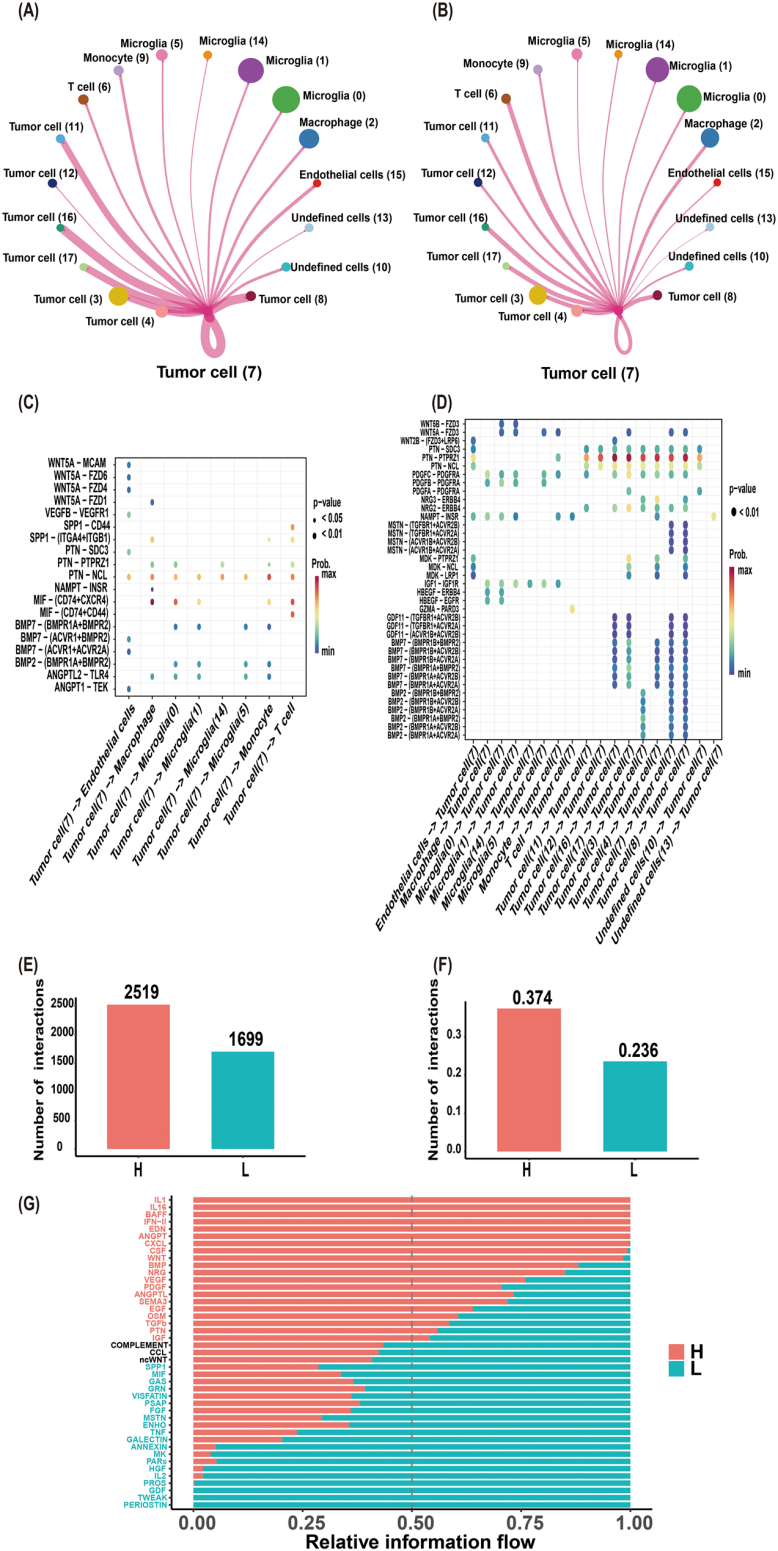
Cluster 7 communication networks and grade-specific interactions in IDH-mutant astrocytoma. (**A**) Interaction frequency between cluster 7 and other clusters. (**B**) Communication intensity of cluster 7 with other clusters. (**C**) Ligand-receptor pairs, when cluster 7 functions as a ligand source. (**D**) Receptor-ligand pairs, when cluster 7 serves as a receptor. (**E**-**F**) Comparative analysis of communication quantity (**E**) and strength (**F**) between high-grade versus low-grade astrocytoma. (**G**) High-to-low-grade ratio of characteristic cellular interactions. Tumor cell [7]: cluster 7, which consists of tumor cells. H: high-grade IDH-mutant astrocytoma. L: low-grade IDH-mutant astrocytoma

CellChat analysis revealed that cluster 7 exhibited the most frequent interactions with tumor subpopulations (clusters 8, 16, 11, 17, 3, and 4) (Fig. 6A), while demonstrating particularly strong communication intensity with T-cell and macrophage clusters (Fig. 6B). Notably, these immune interactions were significantly enriched in high-grade IDH-mutant astrocytoma (Supplementary Fig. 3E) compared to their low-grade counterparts (Supplementary Fig. 3F), suggesting cluster 7 may play a key role in modulating the immune microenvironment through crosstalk with T cells and macrophages.

Cluster 7 exhibited distinct ligand-receptor interactions: as a ligand, it primarily engaged the MIF-(CD74 + CXCR4) pathway (Fig. 6C), while as a receptor, it predominantly interacted via PTN-PTPRZ1 (Fig. 6D). Cluster 7-specific analyses revealed strong ANGPTL and MSTN signaling when acting as ligands with non-tumor cells (Supplementary Fig. 3A-B), and prominent EGF (non-tumor cells) and NRG (tumor cells) pathways when functioning as a receptor (Supplementary Fig. 3C- D).

Comparative analysis of cellular communication between high-grade and low-grade IDH-mutant astrocytoma revealed significantly enhanced interaction frequency (Fig. 6E) and intensity (Fig. 6F) in high-grade astrocytoma. Differential heatmaps visualized these disparities, with red indicating pathways amplified in high-grade astrocytoma and blue marking suppressed interactions (Supplementary Fig. 2G-H). The ratio of high-to-low-grade communication further underscored these differences (Fig. 6G).

### Impact of cluster 7 on immunotherapy response in IDH-mutant Astrocytoma

**Fig. 7:**
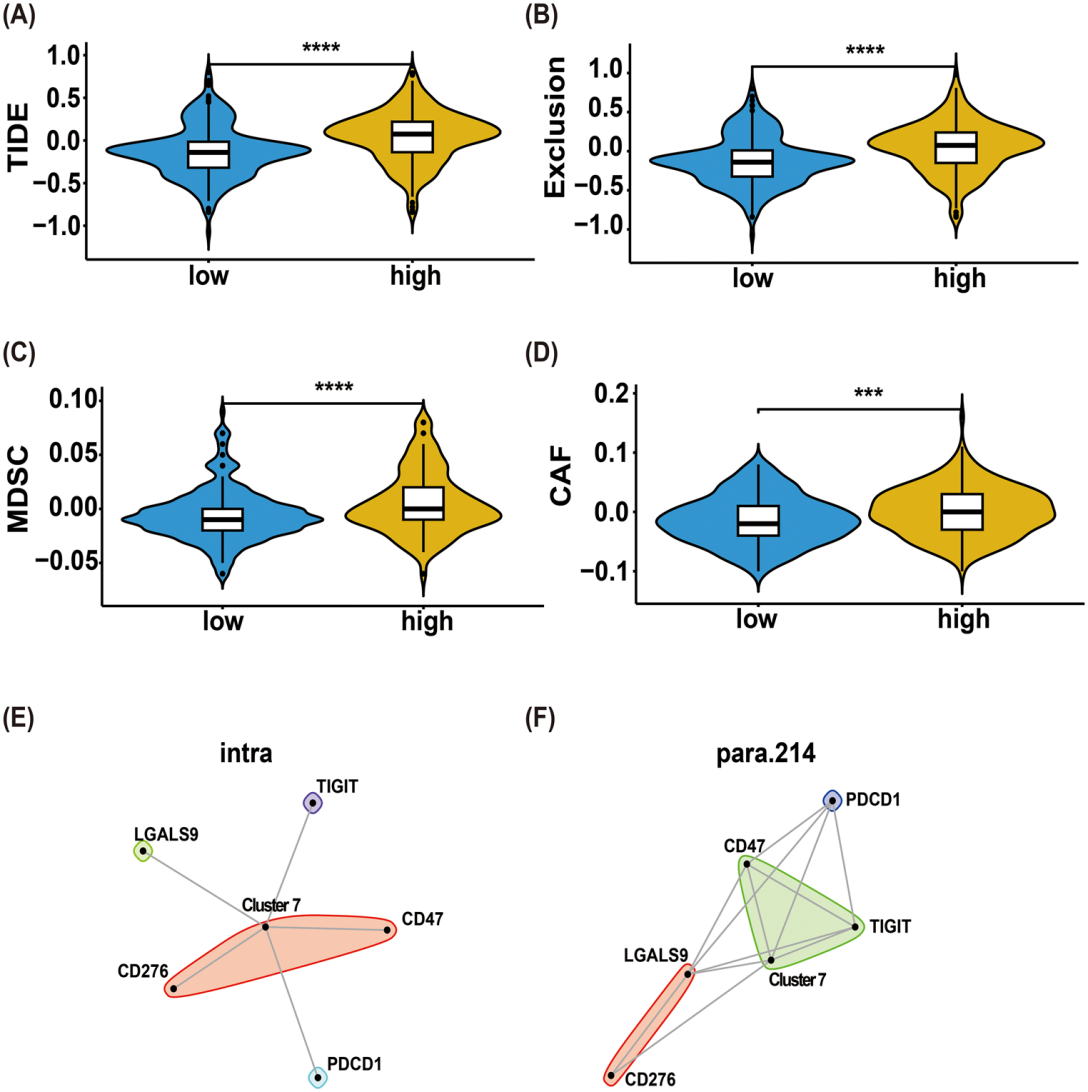
Impact of cluster 7 on immunotherapy response in IDH-mutant astrocytoma. (**A**) TIDE scores, (**B**) Exclusion scores, (**C**) MDSC scores, and (**D**) CAF scores were significantly elevated in the cluster 7-high group compared to the low group, indicating poorer immunotherapy response. (**E**-**F**) Spatial co-localization of cluster 7 with immune checkpoint genes (*TIGIT*,* LGALS9*,* CD47*,* CD276*,* PDCD1*) was observed in both *intra*-tumoral (**E**) and para-tumoral (**F**) regions. TIDE: Tumor Immune Dysfunction and Exclusion. MDSC: Myeloid-derived suppressor cells. CAF: Cancer-associated fibroblasts. ***: *p* < 0.001; ****: *p* < 0.0001

To assess the immunomodulatory role of cluster 7, we performed TIDE analysis on a combined cohort of 429 IDH-mutant astrocytoma (184 WHO grade 2, 201 grade 3, and 44 grade 4) from TCGA and CGGA. Using GSVA, samples were stratified into cluster 7-high and 7-low groups based on median expression of the top 100 signature genes (Supplementary Data). The cluster 7-high group exhibited significantly elevated TIDE scores (Fig. 7A), along with higher Exclusion (Fig. 7B), MDSC (Fig. 7C), and CAF scores (Fig. 7D), collectively indicating poorer immunotherapy responsiveness compared to the low-expression group.

Spatial transcriptomics further revealed co-localization between cluster 7 and immune checkpoint genes (*TIGIT*,* LGALS9*,* CD47*,* CD276*,* PDCD1*) across both in situ (Fig. 7E) and para-tumoral (Fig. 7F) regions. These findings suggest that cluster 7 enrichment correlates with an immunosuppressive microenvironment and reduced therapeutic efficacy of immune checkpoint blockade.

### Spatial transcriptomics analysis of cluster 7

**Fig. 8:**
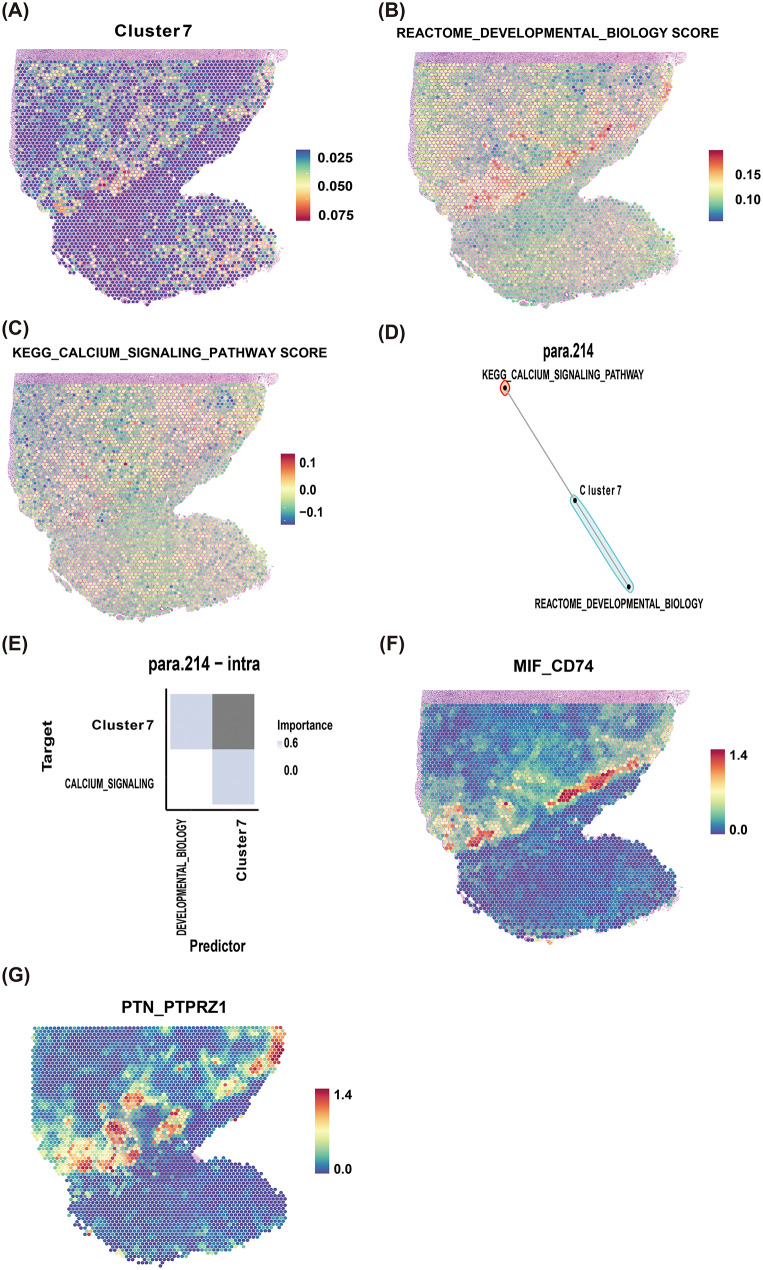
Spatial transcriptomics analysis of cluster 7. (**A**) Spatial distribution of cluster 7. (**B**-**C**) Spatial patterns of REACTOME_DEVELOPMENTAL_BIOLOGY (**B**) and KEGG_CALCIUM_SIGNALING_PATHWAY (**C**). (**D**-**E**) Co-localization of cluster 7 with both pathways in para.214 (**D**) and dual (para.214 + intra) perspectives (**E**). (**F**-**G**) Spatial mapping of ligand-receptor pairs MIF_CD74 (**F**) and PTN_PTPRZ1 (**G**)

We employed RCTD to spatially map cluster 7 distribution using single-cell-derived reference data (Fig. 8A). Notably, the spatial patterns of REACTOME_DEVELOPMENTAL_BIOLOGY (Fig. 8B) and KEGG_CALCIUM_SIGNALING_PATHWAY (Fig. 8C) exhibited concordance with cluster 7 localization. Spatial co-localization between cluster 7 and these pathways was further validated using mistyR (Fig. 8D-E). Additionally, stLearn analysis confirmed the predominant cell-cell communication axes involving MIF_CD74 (Fig. 8F) and PTN_PTPRZ1 (Fig. 8G) at the spatial transcriptomic level.

## Discussion

Recent years have witnessed the application of single-cell and spatial transcriptome sequencing in glioblastoma research [9–11], yet these techniques remain underutilized in the study of IDH-mutant astrocytoma. Notably, some investigations have combined astrocytoma and oligodendroglioma under the umbrella term “IDH-mutant gliomas” [4], an approach that differs fundamentally from studies specifically focused on IDH-mutant astrocytoma. Furthermore, certain studies employ the 2016 WHO Classification of Tumors of the Central Nervous System, a practice that may compromise the precision of their findings [36].

We stratified IDH-mutant astrocytoma cases—which underwent meticulous preoperative planning, precise intraoperative resection, and postoperative pathological confirmation—into low-grade and high-grade groups. This stratification facilitates the investigation of mechanisms underlying tumor progression from low-grade to high-grade disease. Single-cell RNA sequencing analysis identified a distinct *GSX1*^+^ cluster (Cluster 7) enriched in high-grade IDH-mutant astrocytoma. In both TCGA and CGGA (two independent cohorts), this cell population was strongly associated with poor prognosis and emerged as an independent prognostic factor, indicating that patients harboring Cluster 7 tumor cells exhibit worse clinical outcomes. The transcription factor GSX1 emerges as a pivotal regulator of neural progenitor dynamics across developmental contexts. While Pei et al. established its role in promoting neuronal differentiation by antagonizing *GSX2* in the telencephalon [37]. Ma et al. further revealed that its expression is tightly controlled by BMP/SMAD signaling gradients, ensuring orderly temporal fate transitions in the cerebellum [38]. Notably, *GSX1*’s functional versatility extends beyond development: Bergeron et al. implicated *GSX1*^+^ neurons in sensorimotor gating circuits, with loss of *GSX1* disrupting prepulse inhibition—a hallmark of neuropsychiatric disorders [39]. Together, these studies underscore *GSX1* as a molecular nexus linking extrinsic signaling (e.g., BMP), intrinsic transcriptional programs, and higher-order neural circuitry. Our identification of *GSX1*^+^ clusters in high-grade IDH-mutant astrocytoma aligns with its broader role in fate determination and malignancy, suggesting conserved mechanisms whereby *GSX1* may drive aggressive phenotypes through aberrant differentiation or synaptic signaling pathways. Similarly, recent studies have identified *EGFR* alteration as an additional adverse prognostic marker in IDH-mutant astrocytoma [40].

Pseudotime trajectory analysis positioned Cluster 7 at the terminal stage, suggesting a highly malignant phenotype. Furthermore, cell-cell communication analysis revealed significantly more extensive interaction networks in high-grade tumors compared to low-grade counterparts. Cluster 7 was particularly characterized by prominent MIF_CD74 and PTN_PTPZ1 ligand-receptor pairs, a finding corroborated by spatial transcriptomics. These findings are clinically relevant, as PTN is an independent prognostic marker for glioblastoma and PTN-PTPRZ1 promotes tumor growth [41, 42], while MIF drives immune evasion and tumor progression [43, 44]—suggesting these interactions may contribute to malignant progression in IDH-mutant astrocytoma.

Additionally, Cluster 7 displayed an immunosuppressive signature, with spatial co-localization of multiple immune checkpoint-related genes. This aligns with recent reports of a novel immune/mesenchymal-enriched (IME) subtype in IDH-mutant astrocytoma, defined by gemistocytic differentiation, immune infiltration, and unfavorable prognosis [45]. Pathway analysis further demonstrated that Cluster 7 exhibited marked activation of REACTOME_DEVELOPMENTAL_BIOLOGY and KEGG_CALCIUM_SIGNALING_PATHWAY.

These findings collectively demonstrate that the presence of Cluster 7 signifies a highly malignant state in astrocytoma and is associated with poor responsiveness to immunotherapy. This subpopulation is characterized by prominent MIF_CD74 and PTN_PTPRZ1 ligand-receptor interactions, along with marked activation of the REACTOME_DEVELOPMENTAL_BIOLOGY and KEGG_CALCIUM_SIGNALING_PATHWAY.

### Limitation

While our study provides novel insights into the *GSX1*^*+*^ subpopulation in IDH-mutant astrocytoma, several limitations warrant consideration. First, although spatial transcriptomics enabled regional characterization of *GSX1*^*+*^ cluster at the RNA level, the inherent inability to directly assess protein-level interactions may obscure functionally relevant pathways. Future spatial proteomics studies could validate these findings by mapping ligand-receptor interactions and protein network coordination. Second, while computational analyses identified putative ligand-receptor axes (e.g., PTN_PTPRZ1 and MIF_CD74), their mechanistic roles in immunosuppression, malignant progression, and intercellular communication require experimental validation through functional assays. Third, although TCGA/CGGA cohorts revealed prognostic correlations, prospective multicenter studies with standardized treatments are needed to confirm clinical utility. Addressing these gaps through integrated omics approaches and rigorous validation will strengthen the translational significance of our findings.

## Conclusions

In conclusion, our single-cell analysis identified a distinct subpopulation in high-grade IDH-mutant astrocytomas that is preferentially associated with advanced disease stages. This cluster demonstrates molecular characteristics, including enrichment in Developmental Biology and Calcium Signaling pathways, as well as PTN_PTPRZ1/MIF_CD74 interactions, which may contribute to its observed correlation with poorer clinical outcomes. These findings may provide new insights for developing therapeutic strategies against this clinically challenging disease.

## Supplementary Information

Below is the link to the electronic supplementary material.

Supplementary Material 1

Supplementary Material 2

Supplementary Material 3

Supplementary Material 4

Supplementary Material 5

Supplementary Material 6

## Data Availability

The data presented in this study are available on request from the corresponding author.

## References

[1] D.N. Louis, A. Perry, P. Wesseling, D.J. Brat, I.A. Cree, D. Figarella-Branger et al., The 2021 WHO classification of tumors of the central nervous system: a summary. Neuro-Oncology. **23**(8), 1231–1251 (2021) 34185076 10.1093/neuonc/noab106 PMC8328013

[2] L.R. Schaff, I.K. Mellinghoff, Glioblastoma and other primary brain malignancies in adults. Jama 2023; **329**(7) 10.1001/jama.2023.0023 PMC11445779 36809318

[3] K.F. Roberts, S.M. Dahiya, Mitotic index is (still) important for grading isocitrate dehydrogenase (IDH)-mutant Astrocytoma. Neuro-Oncology. **25**(8), 1450–1451 (2023) 37097042 10.1093/neuonc/noad063 PMC10398803

[4] J.J. Miller, L.N. Gonzalez Castro, S. McBrayer, M. Weller, T. Cloughesy, J. Portnow et al., Isocitrate dehydrogenase (IDH) mutant gliomas: A society for Neuro-Oncology (SNO) consensus review on diagnosis, management, and future directions. Neuro-Oncology. **25**(1), 4–25 (2023) 36239925 10.1093/neuonc/noac207 PMC9825337

[5] E.H. Bell, P. Zhang, E.G. Shaw, J.C. Buckner, G.R. Barger, D.E. Bullard et al., Comprehensive genomic analysis in NRG oncology/RTOG 9802: a phase III trial of radiation versus radiation plus procarbazine, lomustine (CCNU), and vincristine in high-risk low-grade glioma. J. Clin. Oncol. **38**(29), 3407–3417 (2020) 32706640 10.1200/JCO.19.02983 PMC7527157

[6] Comprehensive, Integrative Genomic Analysis of Diffuse Lower-Grade Gliomas, N. Engl. J. Med. **372**(26), 2481–2498 (2015) 26061751 10.1056/NEJMoa1402121 PMC4530011

[7] J.E. Eckel-Passow, D.H. Lachance, A.M. Molinaro, K.M. Walsh, P.A. Decker, H. Sicotte et al., Glioma groups based on 1p/19q, IDH, and tertpromoter mutations in tumors. N. Engl. J. Med. **372**(26), 2499–2508 (2015) 26061753 10.1056/NEJMoa1407279 PMC4489704

[8] J.M. Kros, E. Rushing, A.L. Uwimana, A. Hernández-Laín, A. Michotte, M. Al-Hussaini et al., Mitotic count is prognostic in IDH mutant astrocytoma without homozygous deletion of CDKN2A/B. Results of consensus panel review of EORTC trial 26053 (CATNON) and EORTC trial 22033–26033. Neuro-Oncology. 2023; 25(8): 1443-9 10.1093/neuonc/noac282 PMC10398806 36571817

[9] A.T. Yeo, S. Rawal, B. Delcuze, A. Christofides, A. Atayde, L. Strauss et al., Single-cell RNA sequencing reveals evolution of immune landscape during glioblastoma progression. Nat. Immunol. **23**(6), 971–984 (2022) 35624211 10.1038/s41590-022-01215-0 PMC9174057

[10] W. Lingxiang Wu, J. Wu, Z. Zhang, L. Zhao, M. Li, Zhu et al., Natural Coevolution of tumor and immunoenvironment in glioblastoma. Cancer Discov. **12**(12), 2820–2837 (2022) 36122307 10.1158/2159-8290.CD-22-0196 PMC9716251

[11] W. Wang, T. Li, Y. Cheng, F. Li, S. Qi, M. Mao et al., Identification of hypoxic macrophages in glioblastoma with therapeutic potential for vasculature normalization. Cancer Cell. 2024; **42**(5): 815 – 32.e12. 10.1016/j.ccell.2024.03.013 38640932

[12] Y. Hao, T. Stuart, M.H. Kowalski, S. Choudhary, P. Hoffman, A. Hartman et al., Dictionary learning for integrative, multimodal and scalable single-cell analysis. Nat. Biotechnol. **42**(2), 293–304 (2023) 37231261 10.1038/s41587-023-01767-y PMC10928517

[13] C.S. McGinnis, L.M. Murrow, Z.J. Gartner, DoubletFinder, Doublet detection in single-cell RNA sequencing data using artificial nearest neighbors. Cell. Syst. 2019; **8**(4): 329 – 37.e4. 10.1016/j.cels.2019.03.003 PMC6853612 30954475

[14] S. Bandyopadhyay, M.P. Duffy, K.J. Ahn, J.H. Sussman, M. Pang, D. Smith et al., Mapping the cellular biogeography of human bone marrow niches using single-cell transcriptomics and proteomic imaging. Cell. **187**(12), 3120–40e29 (2024) 38714197 10.1016/j.cell.2024.04.013 PMC11162340

[15] M.J. Goldman, B. Craft, M. Hastie, K. Repečka, F. McDade, A. Kamath et al., Visualizing and interpreting cancer genomics data via the Xena platform. Nat. Biotechnol. **38**(6), 675–678 (2020) 32444850 10.1038/s41587-020-0546-8 PMC7386072

[16] Z. Zhao, K.-N. Zhang, Q. Wang, G. Li, F. Zeng, Y. Zhang et al., Chinese glioma genome atlas (CGGA): A comprehensive resource with functional genomic data from Chinese glioma patients. Genom. Proteom. Bioinform. **19**(1), 1–12 (2021) 10.1016/j.gpb.2020.10.005 PMC8498921 33662628

[17] D. Zeng, Y. Fang, P. Luo, W. Qiu, S. Wang, R. Shen et al., IOBR2: multidimensional decoding tumor microenvironment for immuno-oncology research. bioRxiv; 2024. 10.1101/2024.01.13.575484

[18] A. Liberzon, C. Birger, H. Thorvaldsdóttir, M. Ghandi, P. Mesirov Jill, P. Tamayo, The molecular signatures database hallmark gene set collection. Cell. Syst. **1**(6), 417–425 (2015) 26771021 10.1016/j.cels.2015.12.004 PMC4707969

[19] J. Zhang, H. Li, W. Tao, J. Zhou, GseaVis: an R package for enhanced visualization of gene set enrichment analysis in biomedicine. Med. Res. **1**(1), 131–135 (2025)

[20] Van de B. Sande, C. Flerin, K. Davie, De M. Waegeneer, G. Hulselmans, S. Aibar et al., A scalable SCENIC workflow for single-cell gene regulatory network analysis. Nat. Protoc. **15**(7), 2247–2276 (2020) 32561888 10.1038/s41596-020-0336-2

[21] Y. Zhang, B. Zhang, C. Lv, N. Zhang, K. Xing, Z. Wang et al., Single-cell RNA sequencing identifies critical transcription factors of tumor cell invasion induced by hypoxia microenvironment in glioblastoma. Theranostics. **13**(11), 3744–3760 (2023) 37441593 10.7150/thno.81407 PMC10334835

[22] C. Trapnell, D. Cacchiarelli, J. Grimsby, P. Pokharel, S. Li, M. Morse et al., The dynamics and regulators of cell fate decisions are revealed by pseudotemporal ordering of single cells. Nat. Biotechnol. **32**(4), 381–386 (2014) 24658644 10.1038/nbt.2859 PMC4122333

[23] La G. Manno, R. Soldatov, A. Zeisel, E. Braun, H. Hochgerner, V. Petukhov et al., RNA velocity of single cells. Nature. **560**(7719), 494–498 (2018) 30089906 10.1038/s41586-018-0414-6 PMC6130801

[24] S. Jin, C.F. Guerrero-Juarez, L. Zhang, I. Chang, R. Ramos, C.-H. Kuan et al., Inference and analysis of cell-cell communication using cellchat. Nat. Commun. 2021; **12**(1) 10.1038/s41467-021-21246-9 PMC7889871 33597522

[25] P. Jiang, S. Gu, D. Pan, J. Fu, A. Sahu, X. Hu et al., Signatures of T cell dysfunction and exclusion predict cancer immunotherapy response. Nat. Med. **24**(10), 1550–1558 (2018) 30127393 10.1038/s41591-018-0136-1 PMC6487502

[26] R. 1 Sonja Hänzelmann, Castelo, Justin Guinney,. GSVA: gene set variation analysis for microarray and RNA-seq data. BMC Bioinf. 2013 Jan **16**: 14:7 10.1186/1471-2105-14-7 PMC3618321 23323831

[27] D.M. Cable, E. Murray, L.S. Zou, A. Goeva, E.Z. Macosko, F. Chen et al., Robust decomposition of cell type mixtures in Spatial transcriptomics. Nat. Biotechnol. **40**(4), 517–526 (2021) 33603203 10.1038/s41587-021-00830-w PMC8606190

[28] J. Tanevski, R.O.R. Flores, A. Gabor, D. Schapiro, J. Saez-Rodriguez, Explainable multiview framework for dissecting Spatial relationships from highly multiplexed data. Genome Biol. 2022; **23**(1) 10.1186/s13059-022-02663-5 PMC9011939 35422018

[29] D. Pham, X. Tan, B. Balderson, J. Xu, L.F. Grice, S. Yoon et al., Robust mapping of spatiotemporal trajectories and cell–cell interactions in healthy and diseased tissues. Nat. Commun. 2023; **14**(1) 10.1038/s41467-023-43120-6 PMC10676408 38007580

[30] K. Aguilar-Cázarez, D. Pineau, L. Garcia, G.-H. Huang, S.-Q. Lv, H. Duffau et al., A protocol for explant cultures of IDH1-mutant diffuse low-grade gliomas. J. Visualized Experiments. **9**, 219 (2025 May) 10.3791/67260 40418677

[31] E. Karimi, M.W. Yu, S.M. Maritan, L.J.M. Perus, M. Rezanejad, M. Sorin et al., Single-cell Spatial immune landscapes of primary and metastatic brain tumours. Nature. **614**(7948), 555–563 (2023) 36725935 10.1038/s41586-022-05680-3 PMC9931580

[32] A.S. Venteicher, I. Tirosh, C. Hebert, K. Yizhak, C. Neftel, M.G. Filbin et al., Decoupling genetics, lineages, and microenvironment in IDH-mutant gliomas by single-cell RNA-seq. Science 2017; **355**(6332) 10.1126/science.aai8478 PMC5519096 28360267

[33] S.P. Choksi, L.E. Byrnes, M.J. Konjikusic, B.W.H. Tsai, R. Deleon, Q. Lu et al., An alternative cell cycle coordinates multiciliated cell differentiation. Nature. **630**(8015), 214–221 (2024) 38811726 10.1038/s41586-024-07476-z PMC11996048

[34] Y. Fan, R. Zha, T. Sano, X. Zhao, S. Liu, M.D. Woollam et al., Mechanical tibial loading remotely suppresses brain tumors by dopamine-mediated downregulation of CCN4. Bone Res. 2021; **9**(1) 10.1038/s41413-021-00144-2 PMC8144433 34031366

[35] H. Hu, Q. Mu, Z. Bao, Y. Chen, Y. Liu, J. Chen et al., Mutational landscape of secondary glioblastoma guides MET-Targeted trial in brain tumor. Cell. **175**(6), 1665–78e18 (2018) 30343896 10.1016/j.cell.2018.09.038

[36] F. Klemm, R.R. Maas, R.L. Bowman, M. Kornete, K. Soukup, S. Nassiri et al., Interrogation of the microenvironmental landscape in brain tumors reveals Disease-Specific alterations of immune cells. Cell. **181**(7), 1643–60e17 (2020) 32470396 10.1016/j.cell.2020.05.007 PMC8558904

[37] Z. Pei, B. Wang, G. Chen, M. Nagao, M. Nakafuku, K. Campbell, Homeobox genes Gsx1 and Gsx2 differentially regulate telencephalic progenitor maturation. Proceedings of the National Academy of Sciences. 2011; 108(4): 1675-80 10.1073/pnas.1008824108 PMC3029701 21205889

[38] T.C. Ma, K.I. Vong, K.M. Kwan, Spatiotemporal decline of BMP signaling activity in neural progenitors mediates fate transition and safeguards neurogenesis. Cell. Rep. **30**(11), 3616–24e4 (2020) 32187534 10.1016/j.celrep.2020.02.089

[39] S.A. Bergeron, N. Carrier, G.H. Li, S. Ahn, H.A. Burgess, Gsx1 expression defines neurons required for prepulse Inhibition. Mol. Psychiatry. **20**(8), 974–985 (2014) 25224259 10.1038/mp.2014.106 PMC4362800

[40] C.C. Slocum, P. Nguyen, M. Vij, R.L. Yong, J. Samanamud, S. Hiya et al., EGFR alteration is an adverse prognostic factor in IDH-mutant Astrocytoma. Acta Neuropathol. 2025; **150**(1) 10.1007/s00401-025-02928-w PMC12364977 40828325

[41] A.M. Knudsen, B. Halle, O. Cédile, M. Burton, C. Baun, H. Thisgaard et al., Surgical resection of glioblastomas induces pleiotrophin-mediated self-renewal of glioblastoma stem cells in recurrent tumors. Neuro-Oncology. **24**(7), 1074–1087 (2022) 34964899 10.1093/neuonc/noab302 PMC9248408

[42] Y. Shi, Y.-F. Ping, W. Zhou, Z.-C. He, C. Chen, B.-S.-J. Bian et al., Tumour-associated macrophages secrete Pleiotrophin to promote PTPRZ1 signalling in glioblastoma stem cells for tumour growth. Nat. Commun. 2017; **8**(1) 10.1038/ncomms15080 PMC5461490 28569747

[43] Y. Zheng, X. Li, X. Qian, Y. Wang, J.-H. Lee, Y. Xia et al., Secreted and O-GlcNAcylated MIF binds to the human EGF receptor and inhibits its activation. Nat. Cell Biol. **17**(10), 1348–1355 (2015) 26280537 10.1038/ncb3222 PMC4785887

[44] M. Mittelbronn, M. Platten, P. Zeiner, Y. Dombrowski, B. Frank, C. Zachskorn et al., Macrophage migration inhibitory factor (MIF) expression in human malignant gliomas contributes to immune escape and tumour progression. Acta Neuropathol. **122**(3), 353–365 (2011) 21773885 10.1007/s00401-011-0858-3

[45] J. Tang, W. Fan, Y. Ruan, X. Liu, F. Qiu, J. Feng et al., Protein-based classification reveals an immune-hot subtype in IDH mutant Astrocytoma with worse prognosis. Cancer Cell. 2025 Sep. **11**:S1535-6108(25)00363-0. 10.1016/j.ccell.2025.08.006 40939590

