# Supplementary figures and images for: Single-cell and spatial transcriptome sequencing analysis reveals characteristics of a unique subpopulation in high-grade IDH-mutant astrocytoma
Source: Cell Oncol (Dordr). 2025 Dec 29;49(1):8. doi: 10.1007/s13402-025-01139-5 (PMC12748311; doi:10.1007/s13402-025-01139-5)

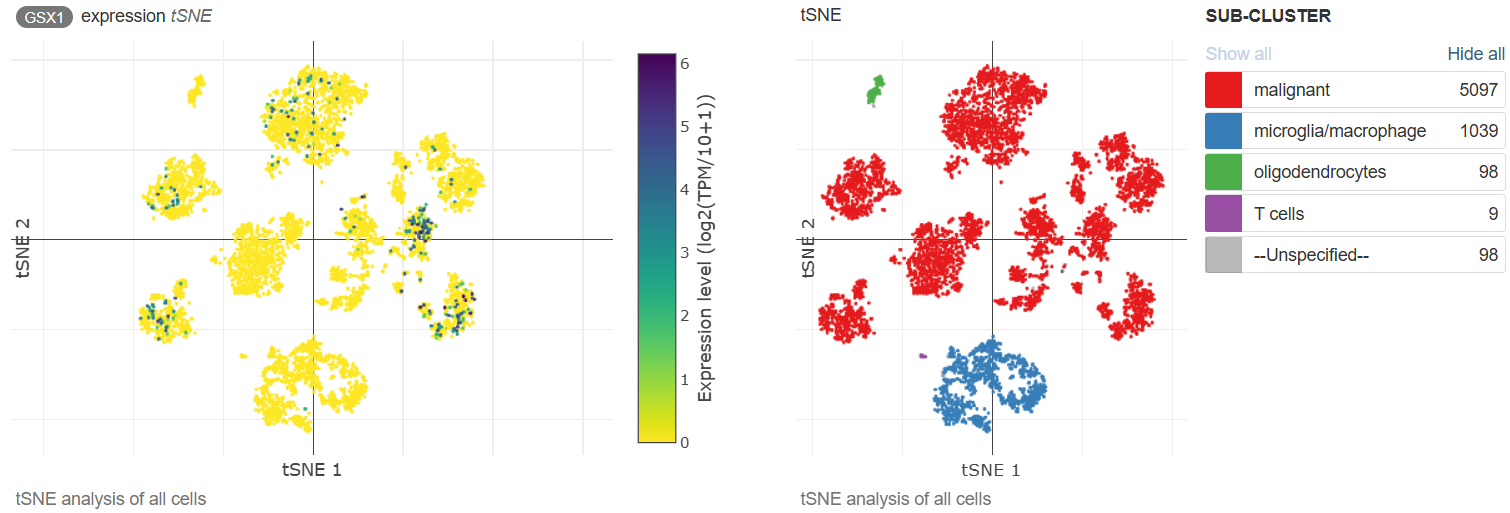

Supplement: Supplementary file 1 — Supplementary Material 1 [file 13402_2025_1139_MOESM1_ESM.png]

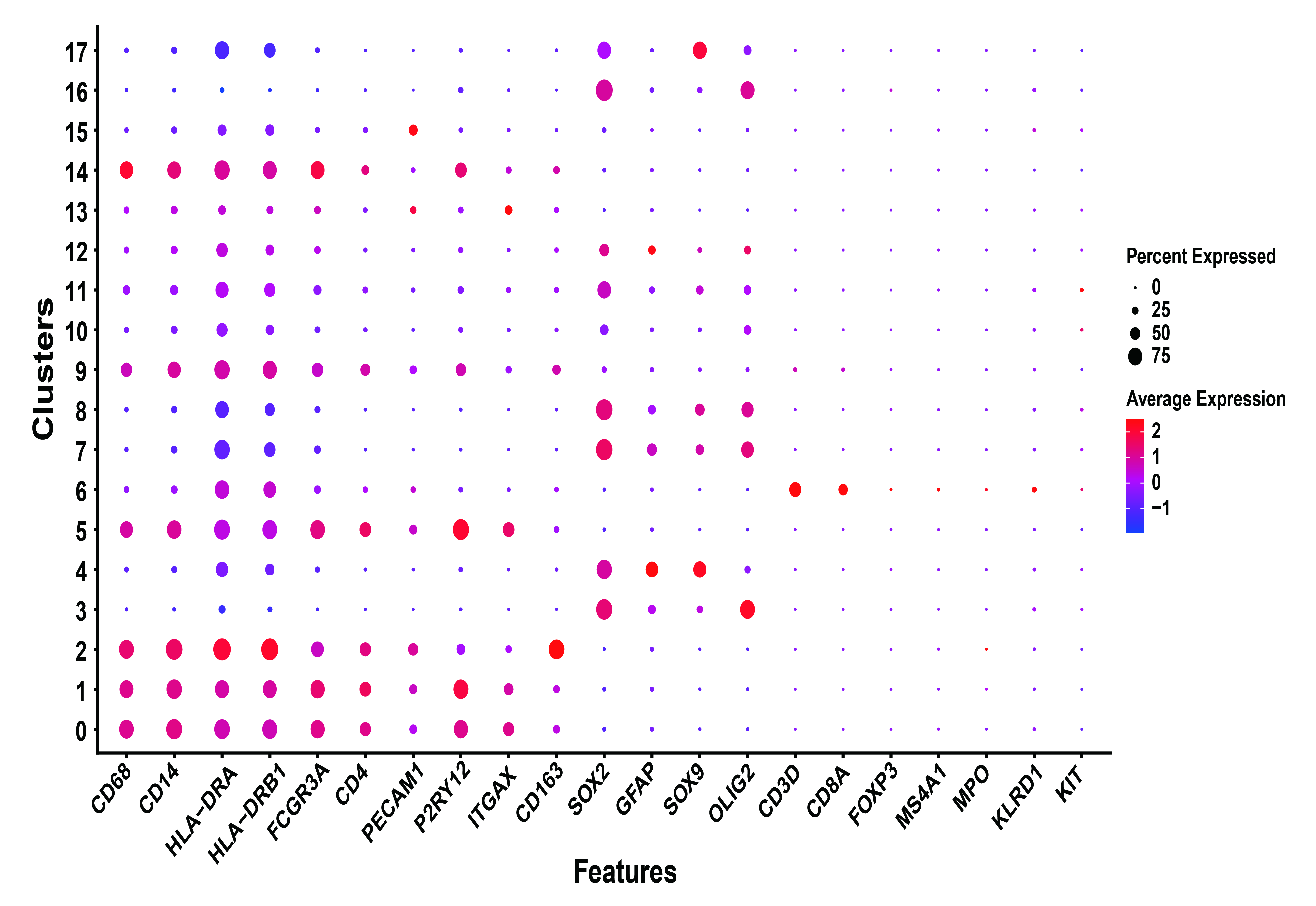

Supplement: Supplementary file 2 — Supplementary Material 2 [file 13402_2025_1139_MOESM2_ESM.tif]

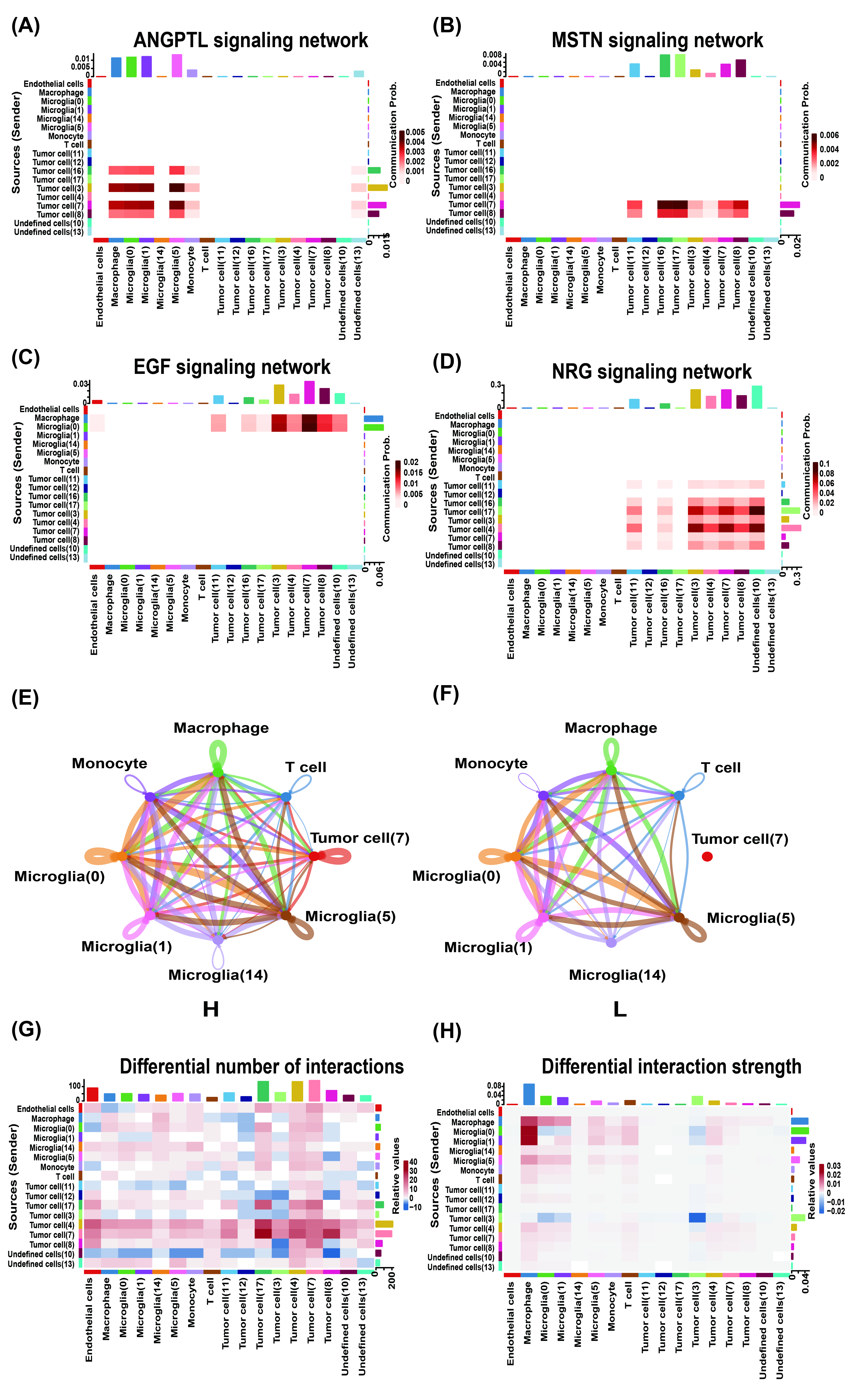

Supplement: Supplementary file 3 — Supplementary Material 3 [file 13402_2025_1139_MOESM3_ESM.tif]
