# Supplementary material for: Single-cell and spatial transcriptome sequencing analysis reveals characteristics of a unique subpopulation in high-grade IDH-mutant astrocytoma
Source: Cell Oncol (Dordr). 2025 Dec 29;49(1):8. doi: 10.1007/s13402-025-01139-5 (PMC12748311; doi:10.1007/s13402-025-01139-5)

1. The original membrane was cut into strips along the molecular weight marker guidelines prior to incubation with specific primary antibodies to allow optimal probing with multiple antibodies of similar molecular weights. The lanes shown here were digitally assembled from the same blot to create a cohesive figure. This assembly process did not alter the original data in any way. As shown in the figure below.


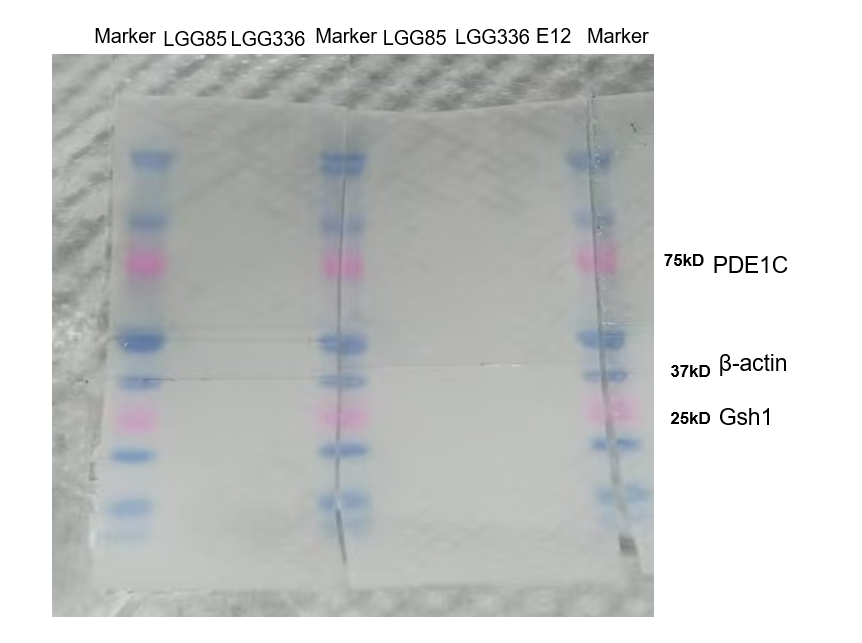


1. Due to the low abundance of GSH1 protein in the experimental samples. The Western Blot detection signal itself is very weak. When piecing together with other protein bands with much higher signal intensity, in order to ensure that the strong signal bands are not overexposed, we must choose a shorter, compromise exposure time. Under this setting, weak bands of GSH1 cannot be displayed. The figure on the left depicts the original full membrane; the figure on the right shows the images obtained from separate exposures.


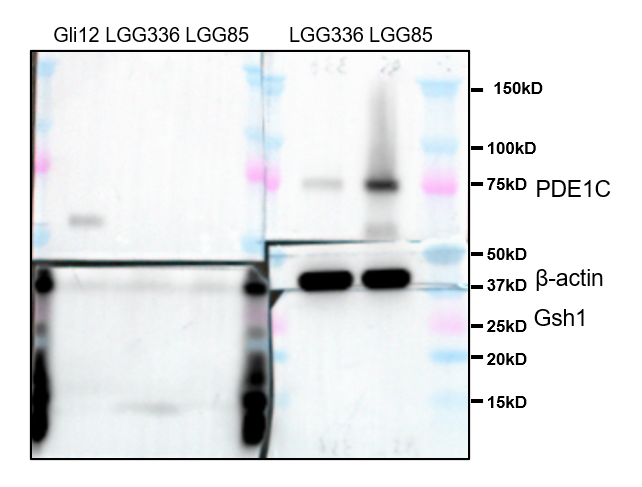

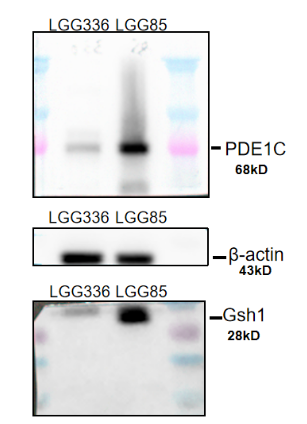

Supplement: Supplementary file 5 — Supplementary Material 5 [file 13402_2025_1139_MOESM5_ESM.docx]
