# Supplementary material for: Single-cell and spatial transcriptome sequencing analysis reveals characteristics of a unique subpopulation in high-grade IDH-mutant astrocytoma
Source: Cell Oncol (Dordr). 2025 Dec 29;49(1):8. doi: 10.1007/s13402-025-01139-5 (PMC12748311; doi:10.1007/s13402-025-01139-5)
Supplement: Supplementary file 6 — Supplementary Material 6 [file 13402_2025_1139_MOESM6_ESM.docx]

**Supplementary Fig. 1** DotPlot showed the cell markers from references to identify the clusters.

**Supplementary Fig. 2** Independent validation in IDH-mutant astrocytoma datasets confirmed *GSX1* expression within the tumor cell population.

**Supplementary Fig. 3 CellChat analysis.** (A) Specific cellular communication between cluster 7 as a ligand and non-tumor clusters. (B) Specific cellular communication between cluster 7 as a ligand and other tumor clusters. (C) Specific cellular communication between cluster 7 as a receptor and non-tumor clusters. (D) Specific cellular communication between cluster 7 as a receptor and other tumor clusters. (E) Cellular communication between cluster 7 and immune clusters in high-grade IDH mutant astrocytoma. (F) Cell communication between immune clusters in low-grade IDH mutant astrocytoma. (G) The difference in communication number between high-grade and low-grade IDH mutant astrocytoma. (H) The difference in communication strength between high-grade and low-grade IDH mutant astrocytoma (red colour indicating stronger communication in high grade compared to low grade, and blue colour indicating weaker communication in high grade compared to low grade). Tumor cell (7): cluster 7, which consists of tumor cells. H: high-grade IDH mutant astrocytoma. L: low-grade IDH mutant astrocytoma.

**Supplementary Data.** Top 100 genes of cluster 7.
